# A first complete phylogenomic hypothesis for diploid blueberries (*Vaccinium* section *Cyanococcus*)

**DOI:** 10.1002/ajb2.16065

**Published:** 2022-10-17

**Authors:** Andrew A. Crowl, Peter W. Fritsch, George P. Tiley, Nathan P. Lynch, Thomas G. Ranney, Hamid Ashrafi, Paul S. Manos

**Affiliations:** ^1^ Department of Horticultural Science North Carolina State University Raleigh North Carolina 27607 USA; ^2^ Department of Biology Duke University Durham North Carolina 27708 USA; ^3^ Botanical Research Institute of Texas Fort Worth Texas 76107 USA; ^4^ Royal Botanic Gardens Kew Richmond TW9 3AE UK; ^5^ Department of Horticultural Science North Carolina State University, Mountain Horticultural Crops Research and Extension Center Mills River North Carolina 28759 USA

**Keywords:** alleles, Ericaceae, homoploid hybridization, HybSeq, phasing, phylogenetics, target enrichment, *Vaccinium*

## Abstract

**Premise:**

The true blueberries (*Vaccinium* sect. *Cyanococcus*; Ericaceae), endemic to North America, have been intensively studied for over a century. However, with species estimates ranging from nine to 24 and much confusion regarding species boundaries, this ecologically and economically valuable group remains inadequately understood at a basic evolutionary and taxonomic level. As a first step toward understanding the evolutionary history and taxonomy of this species complex, we present the first phylogenomic hypothesis of the known diploid blueberries.

**Methods:**

We used flow cytometry to verify the ploidy of putative diploid taxa and a target‐enrichment approach to obtain a genomic data set for phylogenetic analyses.

**Results:**

Despite evidence of gene flow, we found that a primary phylogenetic signal is present. Monophyly for all morphospecies was recovered, with two notable exceptions: one sample of *V. boreale* was consistently nested in the *V. myrtilloides* clade and *V. caesariense* was nested in the *V. fuscatum* clade. One diploid taxon, *Vaccinium pallidum*, is implicated as having a homoploid hybrid origin.

**Conclusions:**

This foundational study represents the first attempt to elucidate evolutionary relationships of the true blueberries of North America with a phylogenomic approach and sets the stage for multiple avenues of future study such as a taxonomic revision of the group, the verification of a homoploid hybrid taxon, and the study of polyploid lineages within the context of a diploid phylogeny.

A ubiquitous component of heathlands and other acidophilic plant communities, as well as a food source for wildlife and humans, the true blueberries (*Vaccinium* section *Cyanococcus* A. Gray; henceforth “*Cyanococcus*”) are of immense ecological and economic value. Commercially cultivated blueberries originate from this group—representing one of only a handful of widely cultivated plants originating in North America. Despite its economic importance, *Cyanococcus* has suffered from conflicting taxonomies with poorly defined species boundaries and little investigation into the evolutionary history of wild populations.

*Cyanococcus* is a reticulate species complex of ca. 9–24 species comprising diploids (2*n* = 2*x* = 24), tetraploids, and hexaploids distributed across much of temperate North America (Figure 1). The section is easily distinguished from other sections of *Vaccinium* L. by several unique or otherwise diagnostic characters (e.g., verrucose branchlets, articulated pedicels, awnless anthers, and pseudo‐10‐locular berries; Camp, 1945; Vander Kloet, 1983). In addition to morphological characters, the available molecular data suggest that the group forms a clade (Kron et al., 2002; A. Crowl et al., unpublished data), although sufficient sampling has yet to be undertaken to satisfactorily test monophyly.

**Figure 1:**
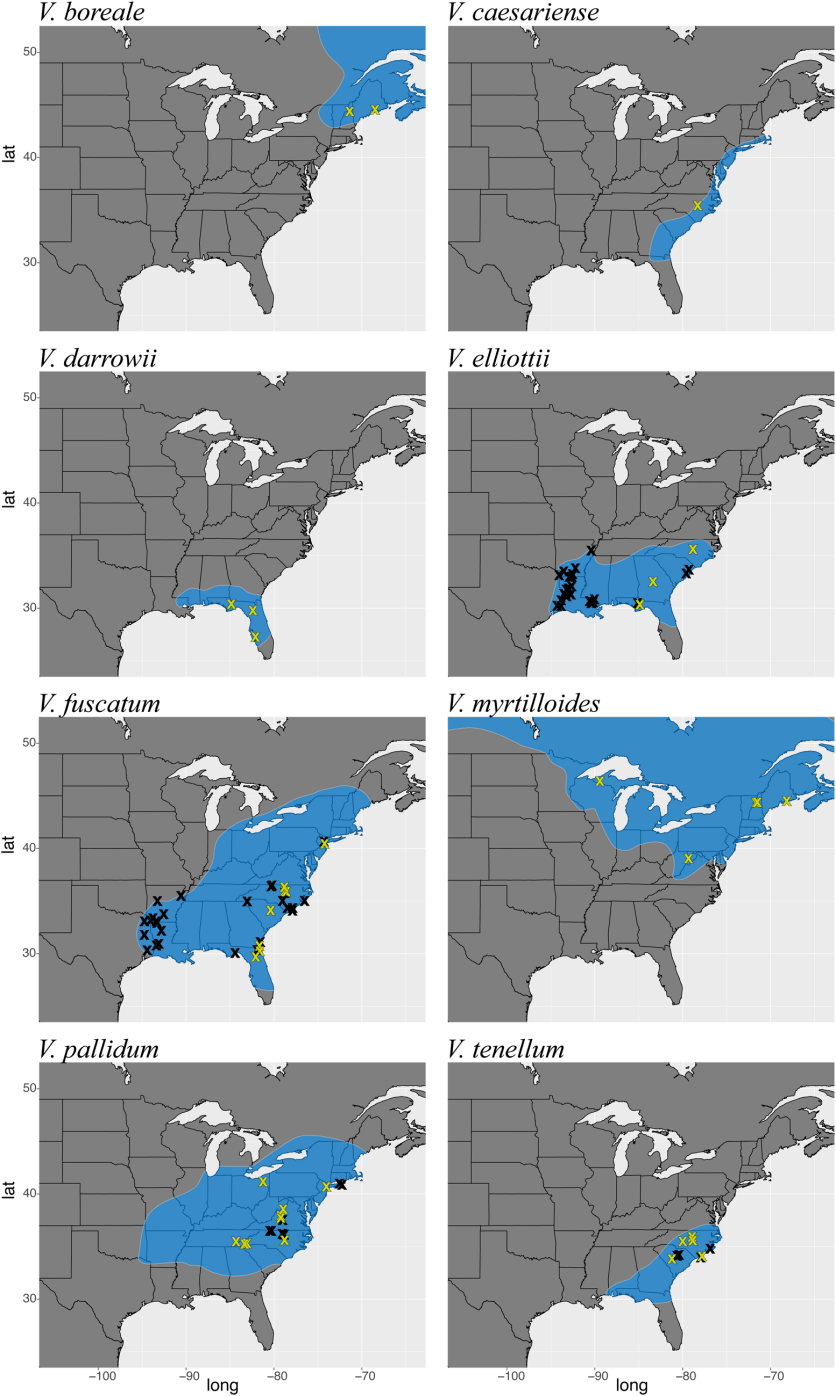
Geographic distribution maps for diploid *Cyanococcus* morphospecies. Black symbols indicate populations included in our broad survey of ploidy and morphology. Yellow symbols indicate a subset of those samples sequenced and included in phylogenomic analyses.

*Cyanococcus* served as a model system during the Modern Synthesis (Huxley, 1942), playing a pivotal role in furthering our understanding of polyploidy and expanding the scope of the movement to include plants. Toward the goal of crop improvement, W. H. Camp and colleagues (Camp, 1942, 1945; Camp and Gilly, 1943; Darrow and Camp, 1945) used data from morphology, crossing studies, genetics, and cytology to propose a complex series of ancestor‐descendant polyploid species relationships in *Cyanococcus*, some through autopolyploidy, others through allopolyploidy. In some cases, Camp (1945) documented size differences correlated with ploidy, such as larger stature and flowers, which has recently been confirmed in one mixed diploid and tetraploid population (Poster et al., 2017). Finally, by equating artificially produced hybrid progeny with morphologically similar plants in the wild, Camp concluded that natural hybrids are rampant among blueberry species, although a strong triploid block, now well known among plant breeders (e.g., Lyrene et al., 2003), was seen to inhibit the viability of progeny with odd‐numbered sets of chromosomes.

Subsequently, S. P. Vander Kloet revised Camp's taxonomy in the context of morphological phenetics. The most consequential of Vander Kloet's conclusions from this work was the supposition that all *Cyanococcus* species >1 m tall (“highbush”) have been derived from a genetic amalgamation of mostly diploid species <1 m tall (“lowbush”), thus forming a “compilospecies” (Harlan and de Wet, 1963) of multiple origins and of variable ploidy (Vander Kloet, 1980, 1983, 1988). In this context, Vander Kloet aggregated 12 of Camp's species into a single highly variable highbush blueberry, *V. corymbosum* L. Although many authors have questioned this extremely broad concept—on the basis of habit; leaf, flower, and stem morphology; phenology; and ecology (e.g., Uttal, 1987; Weakley, 2020; Fritsch et al., in press)—this taxonomic view of *Cyanococcus* is currently considered the standard, having been adopted by the USDA, plant breeders, and many local and regional floras, including the *Flora of North America* (Vander Kloet, 2009).

Much prior research on *Cyanococcus* has highlighted the challenges involved in disentangling this group, but more recent research suggests that the prospects are hopeful for resolving long‐standing questions regarding its species composition, patterns of speciation, and evolutionary history (Fritsch et al., in press). In this respect, the rapid maturation of genomic approaches to the study of complex groups of organisms affords a timely opportunity to revisit the evolution of the true blueberries. The multiple ploidy levels inherent in *Cyanococcus*, the group's ecological and economic importance, and the genomic resources now available make *Cyanococcus* an ideal system for understanding polyploidy and cryptic speciation in flowering plants. Surprisingly, however, the evolution of the group as a whole has yet to be studied with such approaches. This has left *Cyanococcus* in an unsatisfactory state, for both evolutionary biologists and plant breeders alike.

Here, we provide a first glimpse into the evolutionary history of *Cyanococcus* with genomic data by reconstructing a diploid phylogeny with genomic data from hundreds of nuclear loci, with flow cytometry analyses conducted to verify ploidy of all currently recognized putative diploid taxa. Our results will be useful for future study of polyploid *Cyanococcus* lineages and updating the taxonomy of this important group of plants.

## MATERIALS AND METHODS

### Flow cytometry

Ploidy was estimated with flow cytometry at the Mountain Horticultural Crops Research and Extension Center (North Carolina, USA). Leaf samples were quickly dried in the field with silica gel. This dried tissue (~1.5 cm^2^) was finely chopped with a razor blade in a Petri dish with 400 mL of nuclei extraction buffer (CyStain UV Precise P Nuclei Extraction Buffer, Sysmex Partec, Görlitz, Germany). The solution was incubated for 1–2 min at ~24°C and then filtered through Partec CellTrics disposable filters with a pore size of 50 µm to remove tissue debris. Nuclei were stained with 1.6 mL of 4′,6‐Diamidino‐2‐phenylindole (DAPI) staining buffer (CyStain UV Precise P Staining Buffer, Sysmex Partec). Stained nuclei were analyzed with a flow cytometer (Partec PA‐II, Partec) to determine relative genome size. Counts exceeded a minimum of 3000 cells per sample, and two subsamples were run for each sample. Genome sizes were determined by comparing mean relative fluorescence of each sample with an internal standard, *Pisum sativum* L. ‘Ctirad,’ with a known genome size of 8.76 pg (Doležel et al., 2007) and calculated as follows: 2 C genome size of sample = 8.76 pg × (mean fluorescence value of sample/mean fluorescence value of standard). The validity of this method for estimating ploidy levels in *Vaccinium* has been previously demonstrated (with fresh leaf material) by Hummer et al. (2015) and Costich et al. (1993), the latter showing that an observed increase in nuclear DNA content is concurrent with an equivalent increase in ploidy.

### Sampling and sequencing

We sampled 36 *Cyanococcus* individuals, each from different natural populations, representing eight putative diploid species (Appendix S1). Species determination followed the morphospecies concepts summarized in Weakley (2020), in addition to the *V. boreale* I.V. Hall & Aalders concept of Vander Kloet (1988). Three additional taxa—*V. arboreum* Marshall (*Vaccinium* sect. *Batodendron*), *V. macrocarpon* Aiton (*Vaccinium* sect. *Oxycoccus*), and *V. stamineum* L. (*Vaccinium* sect. *Polycodium*)—comprised the outgroup.

DNA extractions were carried out with a modified CTAB approach for all samples (Doyle and Doyle, 1987). The concentration of DNA from extractions was quantified with a Qubit 2.0 (Invitrogen, Carlsbad, California, USA) and the Qubit dsDNA Broad Range Assay Kit following the manufacturer's recommendations. Samples ranging from 115 to 3000 ng of DNA were sent to Arbor Biosciences (Ann Arbor, Michigan, USA) for library preparation and DNA sequencing on a NovaSeq S4 sequencer (Illumina, San Diego, California, USA) with 2 × 150 bp chemistry. The Angiosperms353 v1 target capture kit (Johnson et al., 2019) was used for targeted enrichment of each sample.

### Sequence data processing

Raw sequences were filtered and processed with the Trim Galore wrapper script (version 0.6.5), which uses Cutadapt (version 2.6; Martin, 2011) and FastQC (version 0.11.9; Andrews, 2010) to trim adapters and low‐quality reads based on a given Phred quality score cutoff (‐q 20). Consensus read assembly for target loci was performed with the default settings in HybPiper (version 1.3.1; Johnson et al., 2016). Following the recommendations of McLay et al. (2021), we included available Ericales sequences in the target reference file in addition to the standard Angiosperms353 targets to improve the recovery of targeted loci. Supercontig sequences were then assembled with the *intronerate.py* script available as a part of HybPiper. To screen for potential paralogs, we identified loci/samples in which multiple contigs were generated during the assembly step with the *paralog_investigator.py* script. All loci in which a paralog was suspected were removed from the data set. The remaining consensus reads were used as the reference to generate both IUPAC and allele data sets (see below).

### Allele phasing

HybSeq data are typically processed in a way that results in single consensus sequences for loci, thus ignoring allelic variation (Andermann et al., 2018; Tiley et al., 2021 [preprint]). However, allelic data may be important in the estimation of species networks when gene flow among taxa is present (Tiley et al., 2021 [preprint]). To include this variation, we employed the recently developed bioinformatics pipeline PATÉ (Tiley et al., 2021 [preprint]) to phase alleles. The pipeline uses consensus loci (in this case, supercontig sequences) created with HybPiper as reference sequences, and Illumina reads are mapped back to these loci using the BWA‐MEM algorithm from BWA (Li and Durbin, 2009). Variant calling is carried out at the ploidy level determined by flow cytometry for each individual using the HaplotypeCaller program from GATK (McKenna et al., 2010). Potentially erroneous variant calls are filtered out, based on the following parameters outlined in DePristo et al. (2011), with the VariantFiltration program in GATK: (1) QD < 2.0, (2) FS > 60.0, (3) MQ < 40.0, (4) ReadPosRankSum < 8.0. We also remove variants present on <5% or >95% of reads (AF < 0.05 || AF > 0.95) and variants with a depth of <10 reads (DP < 10). The resulting vcf file for each individual is passed to H‐PoPG (Xie et al., 2016) for allele phasing, which solves for the specified number of haplotypes that minimizes the number of switch errors among the reads present in the BAM file using a dynamic programming solution. PATÉ then takes variants from the largest phase block, combines them with sequences from regions of the locus that could not be phased because of insufficient read overlap, and replaces them with ambiguity codes so that the resulting alleles are the same length as the original consensus loci, similar to previous phasing strategies exclusive to diploids (Kates et al., 2018). PATÉ additionally provides full IUPAC sequences in which all heterozygous sites are replaced by ambiguity codes, which were analyzed alongside individual allele sequences.

### Maximum likelihood analyses

Alignments were carried out with FSA (Bradley et al., 2009). To reduce potential issues with missing data and poorly aligned ends, we removed alignment columns containing >50% missing data. Individual IUPAC gene trees and allele trees were constructed with IQ‐TREE (version 1.6.9; Nguyen et al., 2015). ModelFinder Plus was used to first select the best model for each locus. To assess topological support, we implemented the ultrafast bootstrap approximation UFBoot2 (Hoang et al., 2018) with 1000 replicates in which sites within partitions (loci) were resampled, an approach that is similar to the standard nonparametric bootstrap.

A concatenated alignment was produced for the IUPAC data set with the *pxcat* command in Phyx (Brown et al., 2017). A partitioned phylogenetic analysis, where partitions were individual loci, was performed with IQ‐TREE. The best‐fit partitioning scheme was chosen with the PartitionFinder algorithm (*‐m TESTMERGE*; Lanfear et al., 2012) implemented in IQ‐TREE. A relaxed clustering algorithm (*‐rcluster* 10; Lanfear et al., 2014) was implemented to consider only the top 10% of partitioning schemes. As above, 1000 ultrafast bootstrap replicates were performed to assess support.

### Species‐tree analyses

Multiple species‐tree methods were used to estimate a diploid species tree for *Cyanococcus*. Singular value decomposition quartet species‐tree estimation (SVDquartets; Chifman and Kubatko, 2014), implemented in Paup* (version 4a142; Swofford, 2002), was run on the concatenated IUPAC data matrix, all possible quartets were evaluated, and support was assessed with 100 bootstrap replicates. We also used ASTRAL‐III (version 5.5.6; Zhang et al., 2018) on the individual IUPAC gene trees and allele trees. Alleles were assigned to individuals or species with the allele mapping (‐a) option. We additionally used STACEY (Jones, 2017), available as part of the BEAST2 package (Bouckaert et al., 2014), to estimate a species tree from the IUPAC and allele data sets in a Bayesian framework. Substitution models, clock models, and gene trees were unlinked for all loci. The birth‐death‐collapse model was used as a species‐tree prior. To enable ambiguous site processing of the IUPAC data set, we manually added *useAmbiguities* = *“true”* to the gene‐tree likelihood priors in the XML file. All analyses were run for 10 million generations, retaining one sample every 10,000 generations, or until convergence of all parameters (ESS values > 200), as assessed with Tracer (version 1.7.2; Rambaut et al., 2018).

### Network analyses

Hybridization is thought to be common in *Cyanococcus* (Camp, 1945; Vander Kloet, 1988). To investigate potential reticulation between diploid taxa, we used a pseudolikelihood approach as implemented in SNaQ (Solís‐Lemus and Ané, 2016). For each data set (IUPAC and alleles), we tested models in which we allowed a maximum of zero to three hybridization events (*hmax* = 0–3) and used the log pseudolikelihood profile of these runs to estimate the best‐fitting model. Gene trees inferred from IQ‐TREE were used as input. Twenty independent runs were used for each *hmax* value. The computational constraints of this method precluded the estimation of a network with every sample represented as a tip in the tree. Instead, alleles from individual allele trees were assigned to species, resulting in a network in which tips represented species. The IUPAC data set was subsampled such that each species was represented by one to three samples. To more precisely estimate the placement of the hybrid event suggested by these analyses (i.e., was a single *V. pallidum* population involved or did the hybrid event predate all sampled *V. pallidum* populations?), we constructed an additional IUPAC data set including all eight sampled individuals of *V. pallidum*.

### Concordance‐discordance analyses

Because high bootstrap support can be recovered from phylogenetic analyses despite a low number of genes supporting the topology (e.g., Minh et al., 2020), we additionally assessed conflict within our data set using gene concordance factors (gCF; percentage of genes supporting a given clade) and site concordance factors (sCF; percentage of informative sites) as implemented in IQ‐TREE. Individual IUPAC gene trees were used to calculate both gCF and sCF with 1000 random quartets in the sCF analysis (–scf 1000) for each of the topologies inferred from concatenated and species‐tree analyses (see above).

Discordance was additionally assessed with PhyParts (version 0.0.1; Smith et al., 2015). The best individual IUPAC gene trees inferred from IQ‐TREE were rooted and outgroup taxa were removed with Phyx. Results from these analyses were visualized with the *PhyPartsPieCharts* script. As in the gCF/sCF analyses, we tested each of the topologies inferred from concatenated and species‐tree analyses.

## RESULTS

### Flow cytometry

Flow cytometry analysis of silica‐dried leaf material provided clear genome‐size estimation for 33 of 36 *Cyanococcus* samples (Appendix S1). Average 2 C values ranged from 1.08 to 1.65 pg, within the range for diploid *Vaccinium* individuals previously determined by Hummer et al. (2015) and Redpath et al. (2022). Although we are in the process of reassessing the morphological characters traditionally used to define species in *Cyanococcus*, ploidy estimates mostly conformed to expectations based on morphological identification and observations of the size and density of stomata on second‐year branchlets (Fritsch et al., in press). The one conspicuous exception is *V. boreale*, which was nearly indistinguishable on the basis of morphology from its tetraploid counterpart, *V. angustifolium*, although more detailed analysis of stomatal size and density may facilitate identification (Aalders and Hall, 1962).

### Sequence data

Of the 353 loci targeted with the Angiosperms353 probe set, we successfully captured and sequenced 348. Of these, 25 were flagged as potentially containing paralogs. After removing these loci and all columns containing >50% missing data, the final concatenated IUPAC alignment consisted of 323 loci of alignment length 672,737 bp (= characters); 22,421 of the characters were parsimony informative. Individual supercontig gene (and allele) alignments ranged in length from 272 to 7064 bp.

### Maximum likelihood analyses

**Figure 2:**
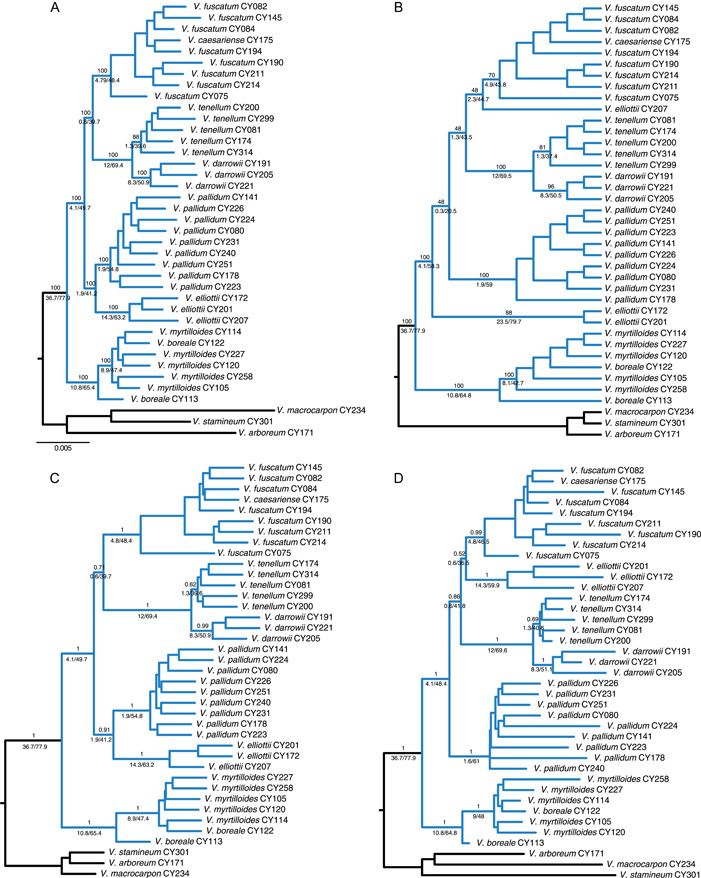
Comparison of topologies recovered from concatenated and species‐tree analyses for the diploid *Cyanococcus* clade (highlighted in blue). Note the inconsistent placement of *V. pallidum* and *V. elliottii* populations between analyses and data sets. Sample numbers refer to the voucher table in Appendix S1. Values above branches indicate support (bootstrap or posterior probability). Values below branches indicate gene concordance factors (gCF) and site concordance factors (sCF). These are reported as gCF/sCF. Intraspecific (population‐level) support values are not shown. (A) Phylogenetic estimate from IQ‐TREE analysis of the concatenated IUPAC data set. (B) Species tree inferred from SVDquartets analysis of the concatenated IUPAC data set. (C) Species tree inferred from ASTRAL‐III analysis of the IUPAC data set. (D) Species tree inferred from ASTRAL‐III analysis of the allele data set.

Concatenated maximum likelihood (ML) analyses of the IUPAC data set with IQ‐TREE resulted in an overall well‐supported topology and maximally supported *Cyanococcus* clade (Figure 2A). A northern lineage of *V. boreale* and *V. myrtilloides* was placed as sister to a large clade composed of the remaining taxa with distributions extending into the southeastern United States. Within this clade, we found three sister‐species relationships: *V. elliottii*–*V. pallidum*, *V. darrowii*–*V. tenellum*, and *V. fuscatum*–*V. caesariense*. This diploid analysis distinguished six maximally supported terminal groups. One sample of *V. boreale* was found to be nested within *V. myrtilloides*, and our only sample of *V. caesariense* nested within *V. fuscatum*.

### Species‐tree analyses

**Figure 3:**
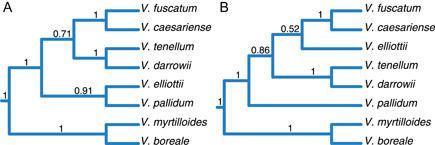
Comparison of species trees inferred from IUPAC and allele data. In both instances, alleles and IUPAC sequences were assigned to species. Note the inconsistent placement of *V. pallidum* and *V. elliottii* between data sets. (A) Species tree inferred from ASTRAL‐III analysis of the IUPAC data set. (B) Species tree inferred from ASTRAL‐III analysis of the allele data set. Values on branches indicate local posterior probability support.

**Figure 4:**
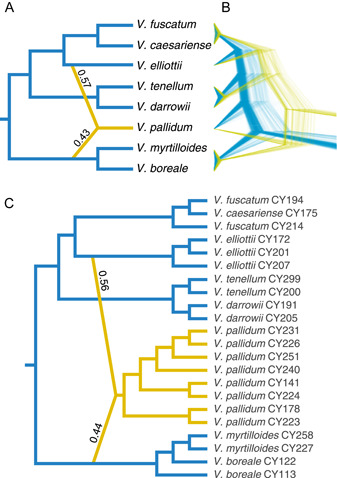
Evidence for the homoploid hybrid origin of *Vaccinium pallidum*. (A) Network inferred from the allele data set in which alleles were assigned to species. Values on hybrid edges are the estimated genomic contributions from each parent (gamma). (B) Posterior distribution of Bayesian species‐tree analysis. The lowest 5% of trees from the posterior distribution are depicted in yellow, showing alternative placement of *V. pallidum* sister to *V. myrtilloides* and *V. boreale*. (C) Network inferred from IUPAC data set with increased population sampling. Note that the hybrid event predates divergence of all sampled *V. pallidum* populations.

The SVDquartets analysis (IUPAC data set) recovered *V. elliottii* as non‐monophyletic, with one sample sister to the *V. fuscatum*–*V. caesariense* clade and the other two in a much deeper position in the tree, albeit with low support (Figure 2B). The remaining relationships were consistent with the results from IQ‐TREE and ASTRAL‐III, including the non‐monophyly of *V. boreale* and the nested position of *V. caesariense* within the *V. fuscatum* clade (Figure 2). ASTRAL‐III analyses recovered a topology (Figure 2C, D) largely consistent with the concatenated ML results. However, the placement of *V. elliottii* differed between IUPAC (Figure 2C) and allele analyses (Figure 2D). This taxon was recovered as sister to *V. pallidum* with the IUPAC data set, whereas it was recovered as sister to other diploid highbush taxa, *V. fuscatum* and *V. caesariense*, with the allele data set, again with low support. This conflicting placement was observed regardless of whether alleles were assigned to individuals (Figure 2) or species (Figure 3). Species‐tree analyses with STACEY placed *V. elliottii* sister to the *V. fuscatum*–*V. caesariense* clade and *V. pallidum* as a stand‐alone lineage. This topology was recovered with both the IUPAC and allele data sets and is consistent with the topology inferred in our ASTRAL analysis of allele data. A unique topology in which *V. pallidum* is sister to the *V. boreale*–*V. myrtilloides* clade was observed when scrutinizing the posterior distribution of trees (Figure 4). This signal, however, is only present in the lowest 5% of the posterior distribution from the IUPAC analysis.

### Network analyses

Network analyses of both the IUPAC and allele data with SNaQ suggested a single hybridization event in our sampling of diploid taxa (Figure 4; Appendix S2). Analysis of the allele data in which alleles were assigned to species recover *V. pallidum* as a hybrid taxon with parental lineages identified as *V. elliottii* and the clade comprising *V. boreale* and *V. myrtilloides* (Figure 4A). Our estimates suggest a nearly equal parental contribution from these two lineages (gamma = 0.57 from *V. elliottii*, and gamma = 0.43 from *V*. boreale–*V. myrtilloides*). Subsequent analysis of the IUPAC data (in which sequences were assigned to samples rather than species) including eight *V. pallidum* individuals confirmed that the hybrid event predates the divergence of all sampled *V. pallidum* populations and that there was a nearly equal genomic contribution from *V. elliottii* (gamma = 0.56) and an ancestor of *V. boreale*–*V. myrtilloides* (gamma = 0.44; Figure 4C).

### Concordance‐discordance analyses

High levels of discordance were found within the IUPAC data set. Despite high bootstrap and posterior probability values, we found relatively low gene (gCF) and site (sCF) concordance factors for the major clades recovered in concatenated and species‐tree analyses (Figure 2). Regarding the inconsistent placement of *V. elliottii*, 1.9% of genes (41% of sites) place it sister to *V. pallidum* whereas 0.6% of loci (36% of sites) support *V. elliottii* as sister to *V. fuscatum*. These results are consistent with those obtained with PhyParts (Appendix S3).

## DISCUSSION

Despite the reputation of *Cyanococcus* as taxonomically intractable, the results of this study, in addition to recent field experience, have led us to agree with Ward (1974) that *Cyanococcus* “is difficult but not in any way an irresolvable tangle of intergrading populations” (p. 192). Although high levels of gene‐tree discordance and topological differences between concatenated ML and species tree methods were observed, the overall topology, monophyly of major clades corresponding to various morphospecies concepts, and placement of these clades were consistent across analyses and data sets. All analyses resolve a northern lineage of *V. boreale* and *V. myrtilloides* sister to the remaining primarily southeastern taxa. Moreover, the analyses consistently recover a close association between *V. darrowii* and *V. tenellum* and between *V. fuscatum* and *V. caesariense*. These results are consistent with an early allozyme study of diploid *Cyanococcus* populations based on phenetic analysis (Bruederle and Vorsa, 1994).

Observed areas of discordance are primarily from inconsistencies in the placement of *V. pallidum* and *V. elliottii*, suggesting hybridization involving these taxa. Network estimation specifically implicated *V. pallidum* as a hybrid taxon. Further analyses including numerous *V. pallidum* individuals sampled across a wide geographic range yielded results showing that the hybrid event predates the divergence of all sampled populations, suggesting that *V. pallidum* is a species of homoploid‐hybrid origin. Parental taxa are suggested to be *V. elliottii* and the lineage giving rise to *V. boreale* and *V. myrtilloides*. A recent study of expressed sequence tag‐polymerase chain reaction markers (Rowland et al., 2021) inferred *V. pallidum* as a close relative of *V. boreale* and *V. myrtilloides*, consistent with this supposition. Although several of our analyses inferred a sister relationship of *V. pallidum* with *V*. *elliottii*, none found *V*. *pallidum* to be sister to the *V*. *boreale*‐*myrtilloides* clade. This signal does, however, appear to be present in our data set when examining the posterior distribution of trees from a Bayesian analysis in STACEY. *Vaccinium pallidum* occupies a geographic range largely overlapping those of its two putative parents (which do not overlap in range), extending farther north than *V. elliottii* and farther south than either *V. boreale* or *V. myrtilloides* (Figure 1). Morphologically, there are not immediately clear characters consistent with the hybrid origin of *V. pallidum*, though this would be expected if the hybrid event was ancient and *V. pallidum* has had sufficient evolutionary time to accumulate morphological attributes distinct from either parent. Moreover, the lack of intermediate morphological characters does not preclude *V. pallidum* as a potential hybrid taxon, because hybridization is not necessarily expected to leave a consistent or predictable phenotypic signature (Anderson, 1948; Rieseberg et al., 1993).

Monophyly for all morphospecies was recovered, with two notable exceptions: *V. boreale* and *V. fuscatum*. One sample of *V. boreale* consistently nested within *V. myrtilloides*, and our *V. caseariense* sample nested within *V. fuscatum* (see also Bruederle and Vorsa, 1994). In the case of *V. boreale*, no evidence of gene flow was detected in our data set, although hybrids of *V. boreale* and *V. myrtilloides* have been reported (Aalders and Hall, 1962). Gene flow was detected between *V. caesariense* and *V. fuscatum* in a suboptimal SNaQ network (not shown), potentially explaining the non‐monophyly of *V. fuscatum*. Alternatively, the long‐standing decision to recognize *V. caesariense* (essentially a glabrous version of *V. fuscatum* occurring on the coastal plain) as an independent entity may be erroneous, and the morphological attributes (i.e., the lack of pubescence on stems and/or leaves) used to distinguish it from *V. fuscatum* may merely be variation within a species. Regarding the *V. corymbosum* “highbush” concept, this result and the apparent sister relationship of *V. elliottii* would appear to at least partially corroborate Vander Kloet's decision to combine these taxa into a single species. The morphologically distinct and phylogenetically cohesive *V. elliottii*, however, challenges this broad concept. Unfortunately, without the inclusion of polyploid taxa we cannot yet satisfactorily address this issue. Furthermore, we have sampled only two populations of *V. boreale* and one population of *V. caesariense* in this study; meaningful conclusions regarding these taxa must await further sampling and more in‐depth analyses.

Although our study of the morphological characters defining species in *Cyanococcus* is ongoing, our working morphospecies concepts for diploid *Cyanococcus* taxa appear to be largely verified with molecular data, as is our hypothesis that the true species composition of this clade likely falls somewhere between the highly divided concept of Camp (1945) and the highly combined concept of Vander Kloet (1988).

### Alleles vs. IUPAC data

Recent studies have attempted to address questions as to the necessity of phasing alleles in phylogenetic reconstruction (e.g., Kamneva et al., 2017; Andermann et al., 2018; Kates et al., 2018; Tiley et al., 2021 [preprint]). We found that in the presence of hybridization, IUPAC and allele data resulted in different topologies. Analyses of IUPAC data consistently inferred a close phylogenetic association between *V. pallidum* and *V. elliottii*, often as sister lineages. Conversely, allele data inferred *V. pallidum* as a lone lineage, phylogenetically intermediate between its two putative parental lineages. This pattern of phylogenetic intermediacy of hybrids in relation to their parents has been previously observed across a wide range of time scales and data types, including morphological data from F_1_ individuals produced through controlled crosses (McDade, 1990), RADseq data from putative naturally formed F_1_ hybrids (Hauser et al., 2017), and target‐enrichment data from taxa involved in ancient introgression events (Crowl et al., 2020). Allele data resolved *V. elliottii* as sister to other “highbush” taxa (i.e., *V. fuscatum* and *V. caesariense*), consistent with our network analyses. This pattern is recovered regardless of whether alleles were assigned to individuals or species. These results suggest that phasing alleles is useful in data sets containing hybrid taxa.

### On homoploid hybrids

Homoploid hybrid speciation is the process by which a new species is formed through hybridization of divergent parent lineages, but without an increase in ploidy (Grant, 1981; Rieseberg, 1997). Although several potential homoploid hybrid species are known in various plant groups—for example, *Carex* (Hodel et al., 2022), *Senecio* (James and Abbott, 2005; Brennan et al., 2012), *Iris* (Arnold, 1993; Taylor et al., 2013; Zalmat et al., 2021), *Pinus* (Wang and Szmidt, 1994), *Penstemon* (Wolfe et al., 1998), and *Paeonia* (Pan et al., 2007)—they appear to be somewhat rare in nature (but see Nieto Feliner et al., 2017). Results of the present study suggest that *V. pallidum* is an additional example. While hybridization is well known in *Vaccinium*, to our knowledge this is the first report of a naturally formed homoploid hybrid species in the group.

To further test this supposition, we additionally considered an F_1_ homoploid (diploid) hybrid resulting from a controlled cross between *V. myrtilloides* and *elliottii*. When included in our data set, network analyses correctly inferred the parents of this hybrid plant and an equal genomic contribution from each parent (Appendix S2). Although far from conclusive, this test case serves as a positive control of sorts and provides increased confidence that our genomic data set and analytical approach can accurately identify a homoploid hybrid taxon. We caution, however, that much work is needed to verify these findings, including further sampling of putative parental taxa, tests of reproductive isolation, investigation of niche divergence, and a detailed morphological study.

### What about polyploids?

While our efforts have been focused on the diploid species of *Cyanococcus*, the group contains numerous polyploid lineages. Polyploids, with more than two copies of each chromosome, remain difficult to analyze in a phylogenetic context. The central challenge of analyzing sequence data from polyploids, and especially allopolyploids, lies in identifying divergent homeolog copies from parental taxa. The majority of bioinformatic tools available for processing next‐generation sequence data were developed for diploid organisms and therefore collapse variable homeolog sequences into a single consensus sequence for downstream analysis. For polyploids, this creates chimeric sequences that obscure signals of polyploidy and a polyploid mode of origin. Conversely, allelic data more accurately capture the complex genomic histories of polyploids and allow for the incorporation of divergent signals from polyploid loci into phylogenomic inference, thus distinguishing allopolyploidy from autopolyploidy and identifying parental taxa.

The diploid phylogenetic estimate presented here, in combination with recent advances in phylogenetic network analysis and a recently developed bioinformatics approach to phasing alleles for arbitrary ploidy from target enrichment data (Tiley et al., 2021 [preprint]), provides an exciting opportunity to investigate polyploid *Cyanococcus* taxa and infer parentage and mode of polyploidization in this challenging group.

## AUTHOR CONTRIBUTIONS

A.A.C., P.W.F., H.A., and P.S.M. designed the study. A.A.C., P.W.F., and P.S.M. carried out fieldwork. N.P.L. conducted flow cytometry analyses. A.A.C. ran phylogenomic analyses. All authors contributed to the intellectual content and writing of the manuscript.

## Supporting information


**Appendix S1**. Voucher table.

**Appendix S2**. Comparison of network analyses with different data sets.

**Appendix S3**. Results from concordance/discordance analyses.

## Data Availability

Raw reads are deposited in the NCBI Sequence Reads Archive (BioProject: PRJNA854616). Final DNA alignment and gene‐tree files are available from the Dryad Digital Repository: doi:10.5061/dryad.cc2fqz68x (Consuegra et al., 2020).
