# Supplementary material for: A first complete phylogenomic hypothesis for diploid blueberries (Vaccinium section Cyanococcus)
Source: Am J Bot. 2022 Oct 17;109(10):1596–606. doi: 10.1002/ajb2.16065 (PMC10286767; doi:10.1002/ajb2.16065)
Supplement: Supplementary file 2 — Appendix S2. Comparison of network analyses with different data sets. [file AJB2-109-1596-s004.pdf]

Appendix S2. SNaQ results.

Likelihood values are given for each model tested from three datasets.
The best network selected (indicated with an asterisks) is shown below each table.

**dataset: all diploids (alleles)**	**-loglik**
net0	142.493988
net1*	65.6625044
net2	59.9362875
net3	59.9362875

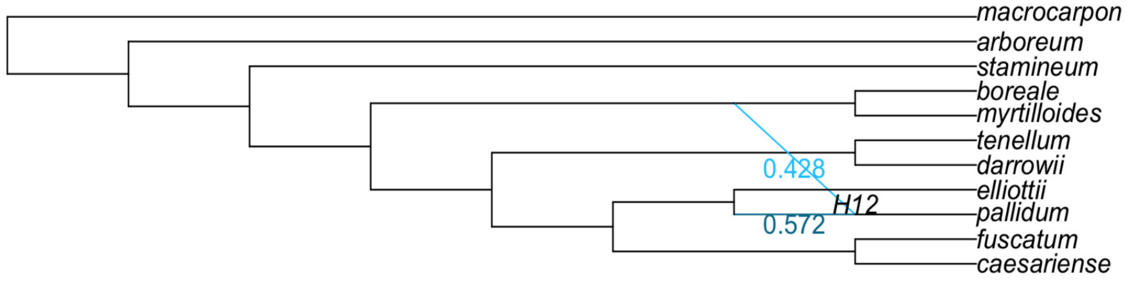

**dataset: all pallidum pops (IUPAC)**	**-loglik**
net0	5292.03115
net1*	2919.78883
net2	2855.42376
net3	2848.98776

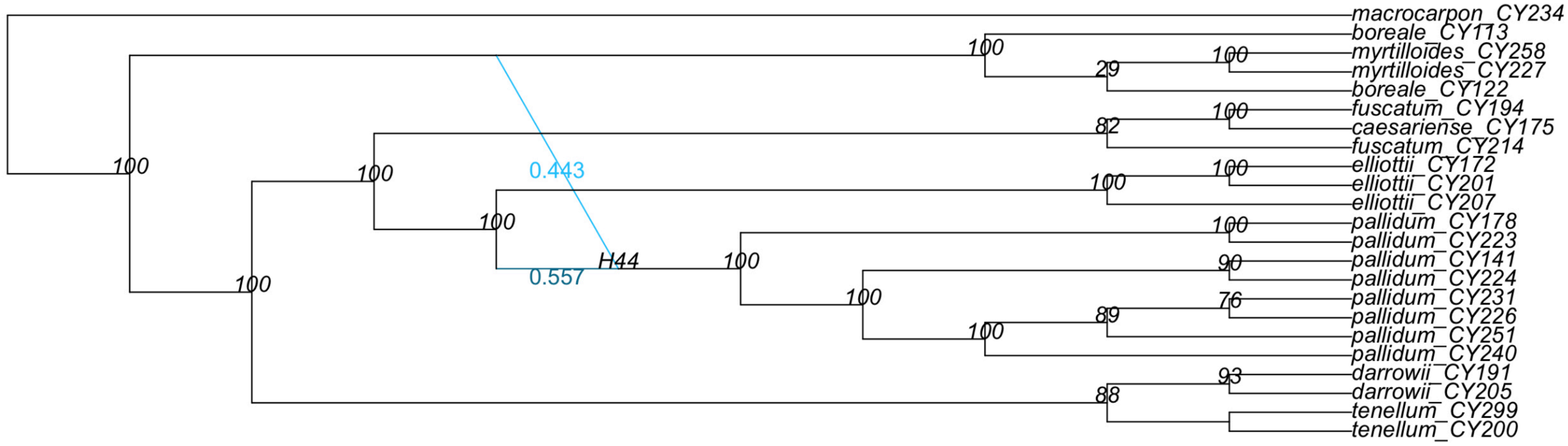

**dataset: F1 myrtilloides x elliottii (alleles)**	**-loglik**
net0	230.960307
net1*	103.464253
net2	90.8944061
net3	90.8944061

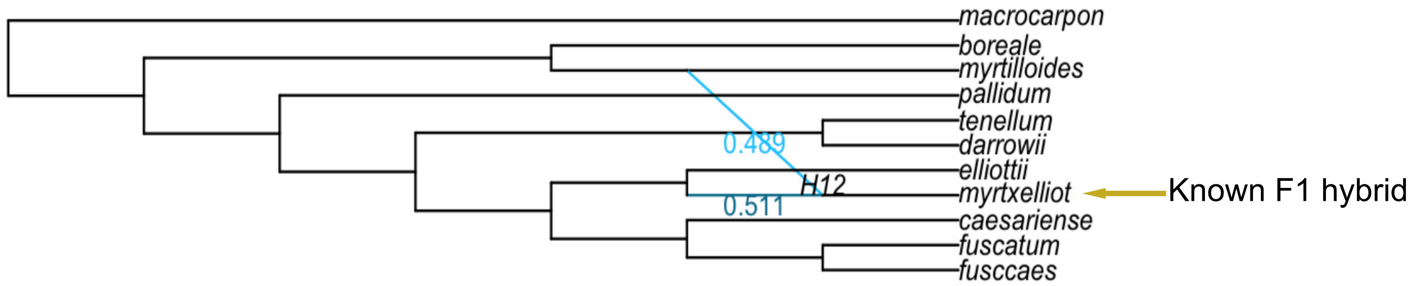

*Network shown for each dataset.
