# Supplementary material for: A first complete phylogenomic hypothesis for diploid blueberries (Vaccinium section Cyanococcus)
Source: Am J Bot. 2022 Oct 17;109(10):1596–606. doi: 10.1002/ajb2.16065 (PMC10286767; doi:10.1002/ajb2.16065)

Appendix S3. PhyParts results.

The best individual IUPAC gene trees inferred from IQ-TREE were used as input to visualize discordance for the four main topologies (A-D) recovered with concatenated and species tree analyses (see also Fig. 2).

(A) iqtree (IUPAC)

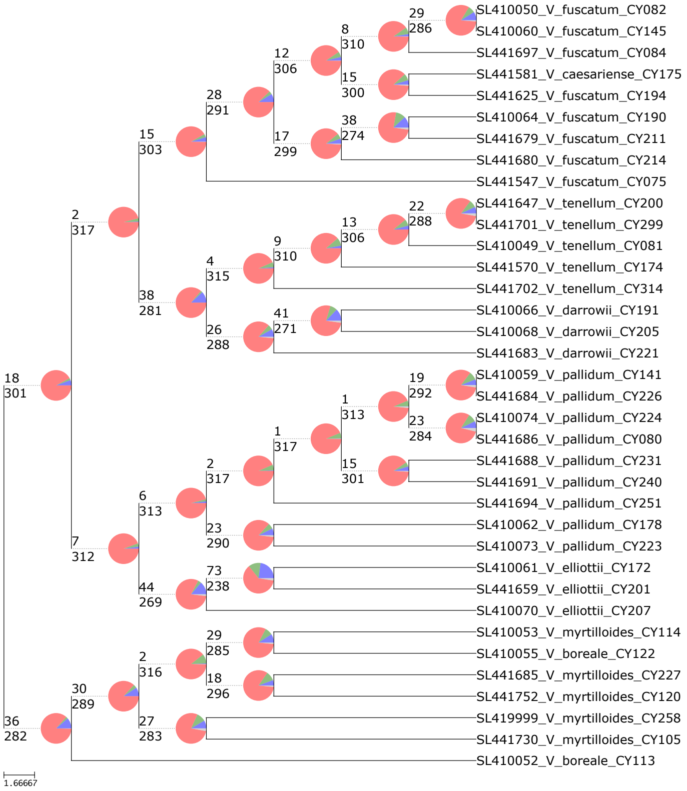

(B) SVDQuartets (IUPAC)

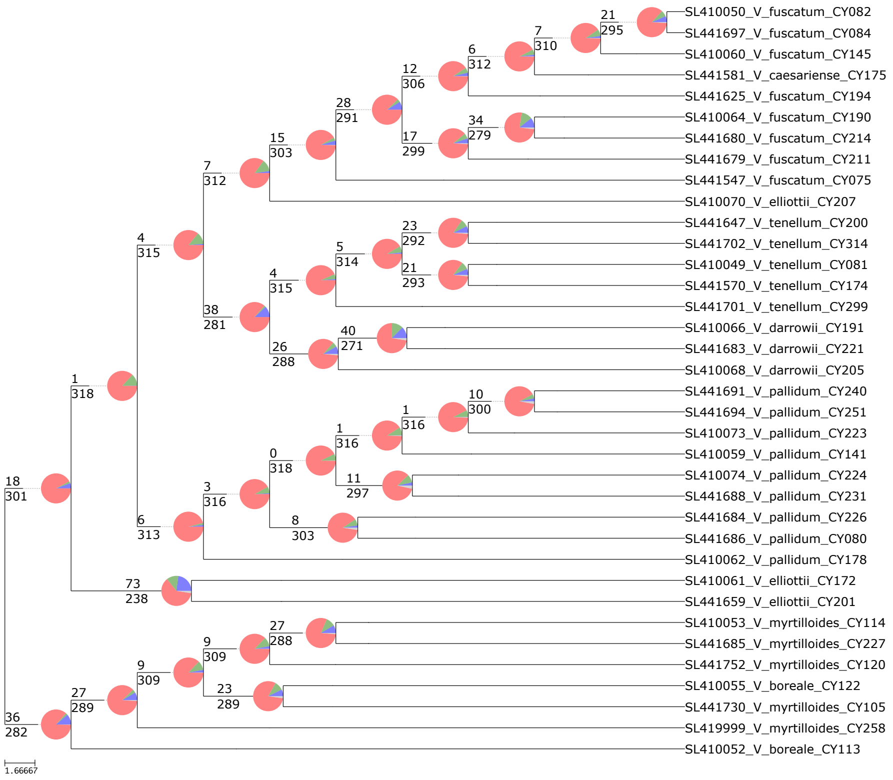

(C) ASTRAL (IUPAC)

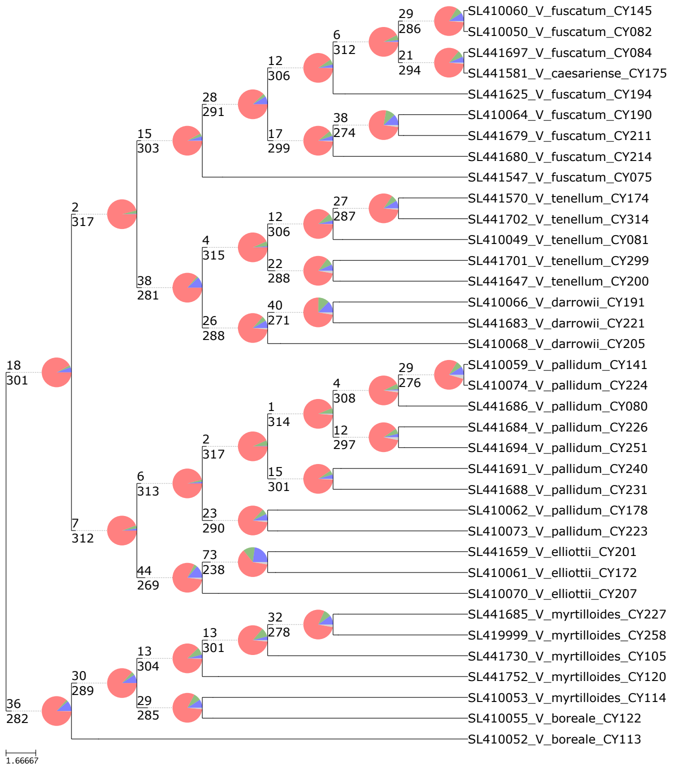

(D) ASTRAL (alleles)

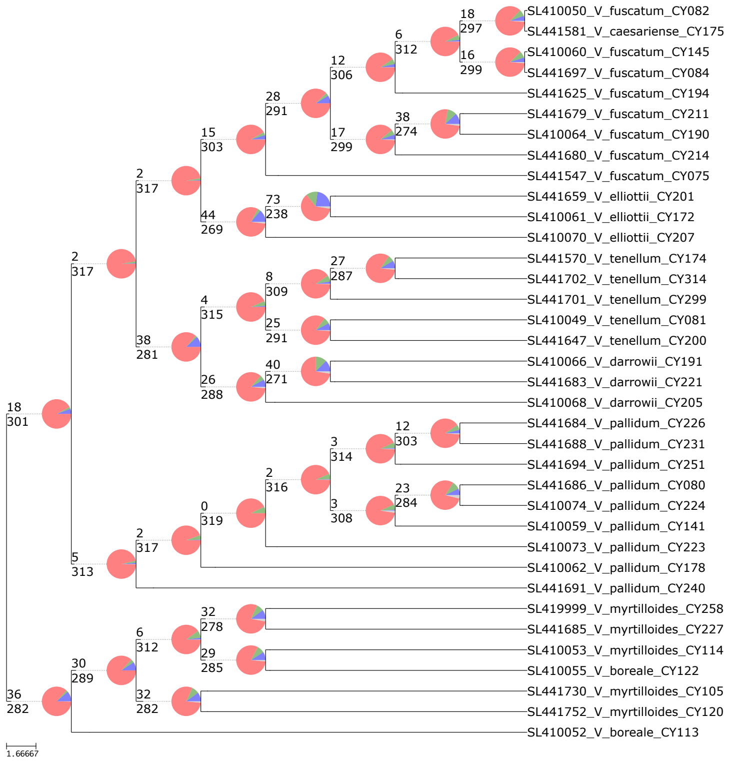

Supplement: Supplementary file 3 — Appendix S3. Results from concordance/discordance analyses. [file AJB2-109-1596-s002.pdf]
