# Supplementary material for: A first complete phylogenomic hypothesis for diploid blueberries (Vaccinium section Cyanococcus)
Source: Am J Bot. 2022 Oct 17;109(10):1596–606. doi: 10.1002/ajb2.16065 (PMC10286767; doi:10.1002/ajb2.16065)
Supplement: Supplementary file 4 [file AJB2-109-1596-s003.docx]

**Supporting Information**

Additional supporting information may be found online in the Supporting Information section at the end of the article.

Appendix S2. Comparison of network analyses with different datasets.

Appendix S3. Results from concordance/discordance analyses.
